# CEACAM 1, 3, 5 and 6 -positive classical monocytes correlate with interstitial lung disease in early systemic sclerosis

**DOI:** 10.3389/fimmu.2022.1016914

**Published:** 2022-10-20

**Authors:** Kana Yokoyama, Hiroki Mitoma, Shotaro Kawano, Yusuke Yamauchi, Qiaolei Wang, Masahiro Ayano, Yasutaka Kimoto, Nobuyuki Ono, Yojiro Arinobu, Koichi Akashi, Takahiko Horiuchi, Hiroaki Niiro

**Affiliations:** ^1^ Department of Medicine and Biosystemic Sciences, Kyushu University Graduate School of Medical Sciences, Fukuoka, Japan; ^2^ Department of Cancer Stem Cell Research, Kyushu University Graduate School of Medical Sciences, Fukuoka, Japan; ^3^ Department of Internal Medicine, Kyushu University Beppu Hospital, Beppu, Japan; ^4^ Department of Medical Education, Faculty of Medical Sciences, Kyushu University, Fukuoka, Japan

**Keywords:** systemic sclerosis, CEACAM, monocytes - cell, interstitial lung disease (ILD), TNF-α - tumor necrosis factor alpha, inflammation

## Abstract

**Background:**

Systemic sclerosis (SSc) is a multiple-organ disease characterized by vascular damage, autoimmunity, and tissue fibrosis. Organ injuries such as interstitial lung diseases (ILD), resulting from inflammatory and fibrosis processes, lead to poor prognosis. Although autoantibodies are detected in the serum of patients with SSc, the mechanisms by which immune cells are involved in tissue inflammation and fibrosis is not fully understood. Recent studies have revealed carcinoembryonic antigen related cell adhesion molecule (CEACAM)-positive monocytes are involved in murine bleomycin-induced lung fibrosis. We investigated CEACAM-positive monocytes in patients with SSc to clarify the role of monocytes in the pathogenesis of SSc.

**Methods:**

The proportion of of CEACAM-positive classical monocytes in healthy controls (HCs) and patients with rheumatoid arthritis (RA) and SSc was evaluated using flow cytometry. The correlation between the proportion of CEACAM-positive monocytes and clinical parameters was analyzed in patients with SSc. Gene expression microarrays were performed in CEACAM-positive and negative monocytes in patients with SSc. Infiltration of CEACAM-positive monocytes into scleroderma skin was evaluated by immunohistochemical staining.

**Results:**

The proportion of CEACAM-positive classical monocytes was increased in patients with early SSc within 2 years after diagnosis, which positively correlated with ESR, serum IgG, and serum KL-6 and negatively correlated with %forced vital capacity. The percentage of CEACAM-positive monocytes decreased after immunosuppressive therapy. CEACAM6-positive cells among classical monocytes were significantly increased in patients with SSc compared with HCs and patients with rheumatoid arthritis. SSc serum induced CEACAM6 expression on monocytes from HCs. Functionally, CEACAM-positive monocytes produced higher levels of TNF-α and IL-1β compared to CEACAM-negative cells and showed activation of the NF-κB pathway. Furthermore, CEACAM6-positive monocytes infiltrated the dermis of SSc.

**Conclusions:**

CEACAM-positive monocytes showed inflammatory phenotypes and may be involved in the tissue inflammation and fibrosis in early SSc. CEACAM-positive monocytes may be one of biomarkers to detect patients with progressive ILD, requiring therapeutic intervention.

## Introduction

Systemic sclerosis (SSc) is a multiple organ disease characterized by vascular damages, autoimmunity, and tissue fibrosis. Multiple organ injuries, such as lung fibrosis, pulmonary arterial hypertension, and gastrointestinal manifestations result in poor prognosis. Interstitial lung disease (ILD) presents progressive course in part of patients with SSc, requiring early therapeutic intervention (1). In addition, intense skin fibrosis and digital ulcers/gangrene impair quality of life. A recent study demonstrated a significant inverse relationship between skin blood perfusion and dermal thickness at the level of dorsum of the middle phalanx of the third fingers in patients with SSc (2), suggesting the interaction between vascular damages and fibrosis. Elucidation of pathology and development of novel methods for early detection of progressive cases are required to modify the disease course of SSc.

The presence of disease-specific autoantibodies such as anti-Scl70, anti-centromere, and anti-RNA polymerase III antibodies, indicates the involvement of the adaptive immune systems in the pathogenesis of SSc; however, the etiology of the disease remains unclear (3–5). Monocytes and macrophages are involved in immune-modulation, inflammation, and tissue-homeostasis and contribute to the pathophysiology of autoimmune and inflammatory diseases (6, 7). These cells express heterogeneous cell surface markers and secrete various cytokines/chemokines that activate and recruit immune and other types of cells into the inflamed tissue. Monocytes are highly plastic and heterogeneous. They exhibit phagocytic activities and differentiation potential into macrophages, dendritic cells, collagen-producing fibrocytes, or myofibroblasts at the site of migration (8). Fibrocytes and myofibroblasts are involved in skin fibrosis in SSc (9, 10). The innate immune system plays a pivotal role in the development of SSc, in addition to the activation of adaptive immunity (11). Circulating monocytes are significantly increased in the peripheral blood of patients with SSc and correlate with poor prognosis and visceral involvement (12). In addition, an altered phenotype of circulating monocytes has been reported in SSc. SSc monocytes have a high potential to differentiate toward type-1 collagen- and α-smooth muscle actin (SMA)-expressing myofibroblasts after stimulation with granulocyte-macrophage colony-stimulating factor, interleukin (IL)-4, and endothelin-1 (13). The production of tissue-inhibitor of metalloproteinase (TIMP)-1 is increased (14) and cell surface expression of CD163, CD204 (15) and Siglec-1 (16) is elevated in SSc monocytes compared to that in monocytes from healthy controls (HCs) (17, 18). CD14-positive monocytes/macrophages are the predominant infiltrating mononuclear cells in skin lesions of patients with recent-onset SSc. Infiltration of CD14+ cells into the heart and lungs was also increased in patients with SSc compared to that in the control tissues. CD14 signals co-localized with collagen-rich lesions in SSc tissues (19). These data indicate that monocytes/macrophages may participate in tissue-inflammatory conditions in the early phase and fibrotic processes in organs during pathological tissue remodeling in SSc.

Transcriptome analysis of CD14+ monocytes from SSc and HCs revealed CD14++CD16- monocytes from SSc expressed significantly higher level of fibronectin than CD14+CD16+, CD14lowCD16+ monocytes from SSc and monocytes from HCs (19). In addition, estimated CD14+CD16- classical monocyte percentages above the mean were associated with shorter lung transplantation-free survival times in idiopathic pulmonary fibrosis, whereas higher percentages of T cells and B cells were not (20). Monocyte count could be incorporated into the clinical assessment of patients with idiopathic pulmonary fibrosis and other fibrotic disorders. These data suggest that CD14+CD16- classical monocytes may be involved in the fibrotic process.

Recent study identified specific subtype of murine monocytes/macrophages involved in bleomycin-induced lung fibrosis. Carcinoembryonic antigen-related cell adhesion molecules (CEACAM)1+Msr1+Ly6C-F4/80-Mac1+ monocytes termed segregated-nucleus-containing atypical monocytes (SatM), share granulocyte characteristics, are regulated by CCAAT/enhancer binding protein β, and required for lung fibrosis by infiltrating into pulmonary interstitium (21). However, human monocytes involved in lung fibrosis in SSc have not been well defined. CEACAM, a subgroup of the CEA family of immunoglobulin-associated proteins, are encoded in the human genome by 12 genes including CEACAM1, CEACAM3-CEACAM8, CEACAM16 and CEACAM18-21. CEACAM1, 3, 5 and 6 work as pathogen receptors which recognize bacteria, virus, and soluble antigens. CEACAM is involved in a variety of processes including cellular growth, immune cell activation, and tissue morphogenesis (22). However, the role of CEACAM in human monocytes is largely unknown. CEACAM1 suppresses apoptosis of monocytes through the phosphatidylinositol-3’ kinase (PI3-kinase) and AKT pathways and functions as a key regulator of contact-dependent control of cell survival, differentiation, and growth (23). Monocytes are increased in patients with SSc and can differentiate into myofibroblasts which produce extracellular matrix. These facts prompted us to investigate whether CEACAM are involved in immune activation and fibrosis monocytes in patients with SSc.

Here, we investigated whether CEACAM-positive monocytes were involved in inflammation and fibrosis using human SSc subjects to clarify the role of these cells in pathogenesis of SSc. The proportion of peripheral blood CEACAM-positive monochytes, especially CEACAM6-positive monocytes were significantly higher in SSc than that in rheumatoid arthritis (RA) or in HCs. This higher percentage of CEACAM-positive monocytes was positively correlated with erythrocyte sedimentation rate (ESR) and serum IgG levels. CEACAM-positive monocytes decreased after the treatment with immunosuppressive agents. CEACAM-positive monocytes produced more TNF-α and IL-1β than CEACAM-negative ones. SSc serum induced CEACAM6-expression on monocytes from HCs. In addition, CEACAM-positive monocytes were associated with progressive interstitial lung disease and were infiltrated in the inflamed skin. Therefore, CEACAM-positive monocytes show inflammatory phenotypes and may be involved in the tissue inflammation and fibrosis in early SSc. These cells may be one of useful markers to detect progressive patients requiring therapeutic intervention. In addition, these cells may be one of therapeutic targets in early SSc. Our data provide new insights into the role of CEACAM-positive monocytes in the pathogenesis of SSc.

## Materials and methods

### Patients and controls

Peripheral blood samples from 34 HCs, 20 patients with RA, and 30 patients with SSc [SSc; cutaneous SSc (lcSSc), n=12; diffuse cutaneous SSc (dcSSc) n=18] were obtained at Kyushu University Hospital. Skin biopsy specimens were collected from five patients with early SSc and two HCs. The diagnosis of SSc was based on the classification criteria of the ACR/EULAR2013 (24). Demographic clinical data of patients with SSc is indicated in **Table 1**. Part of patients were inpatients. All inpatients were required for aggressive therapeutic intervention. This study was approved by the ethics committee of Kyushu University Hospital (approval number 2019-445 and 30-341) in accordance with the Helsinki Declaration. All participants gave written informed consent.

**Table 1:** Demographic clinical data of patients with SSc.

	SSc within 2 years after diagnosis (n = 12)	SSc over 2 years after diagnosis (n = 21)	*p* value
Age, years	50.1 ± 12.9	55.1 ± 12.6	0.27
Female, *n* (%)	6 (50)	16 (76)	0.12
Disease duration, median(IQR), years	0.40 (0.11-0.97)	4.7 (3.0-17.6)	<0.0001
Diffuse SSc, *n* (%)	10 (83)	13 (62)	0.22
Interstitial pneumonia, *n* (%)	8 (67)	16 (76)	0.69
Pulmonary arterial hypertension, *n* (%)	1 (8.3)	7 (33)	0.21
modified Rodnan skin score	20.3 ± 9.0	13.3 ± 11.2	0.21
Autoantibodies			
Anti-Scl70 antibody positive, *n* (%)	6 (50)	6 (30)	0.26
Anti-centromere antibody positive, *n* (%)	1 (8.3)	5 (25)	0.38
Anti-RNA polymeraseIII antibody positive, *n* (%)	2 (17)	2 (10)	0.61
Anti-RNP antibody positive, *n* (%)	2 (17)	6 (29)	0.68
Medications			
Corticosteroid use, *n* (%)	5 (42)	10 (45)	0.74
Immunosuppressive agent use, *n* (%)	3 (25)	2 (10)	0.33

### Flow cytometry and cell sorting

To analyze the proportion of CEACAM+ monocytes, peripheral blood mononuclear cells (PBMCs) were separated by Lymphoprep (STEMCELL Technologies) and stained with the following antibodies according to the manufacturer’s instructions: anti CD14-APC (Clone M5E2, BioLegend #301808), CD16-FITC (Clone 3GB, BioLegend #302006), CD56-Percp/Cy5.5 (Clone HCD56, BioLegend #318322), CEACAM-BV421 (Clone B1.1/CD66, BD Biosciences #562741), CEACAM1-PE (Clone 283340, R&D Systems #FAB2244P), CEACAM3-PE (Clone 06, Sino Biological #11933-H08), CEACAM5-FITC (Clone C365D3, Bio-Rad #MCA1744FT), and CEACAM6-APC (Clone 439424, R&D Systems #MAB3934-SP). Anti-CEACAM mAb (Clone B1.1) recognizes CD66a (CEACAM1), CD66c (CEACAM6), CD66d (CEACAM3) and CD66e (CEACAM5). Data were acquired on a flow cytometry and analyzed using Flowjo v10.6.2 software (BD Biosciences). To isolate CEACAM+ and CEACAM- monocytes, CD14+ monocytes purified with CD14 MicroBeads (Miltenyi) were stained with antibodies; the target cell populations were sorted by flow cytometry, and purity was checked. Sorted cells were lysed in RLT buffer (QIAGEN), and RNA was extracted and used for gene expression microarray analysis (described below).

### Mass cytometry

PBMCs from HCs and patients with SSc were stimulated with 100ng/mL of lipopolysaccharide (LPS; *In vivo*Gen) for 5h and stained with antibodies (shown in **Table 2**) according to the manufacturer’s instructions. The cells were resuspended in 1 µM cisplatin (Fluidigm #201198) to label the non-viable cells and incubated for 5 min at RT. The cells were washed with staining buffer (Fluidigm #201068), and a 5 µL Fc blocking solution (Miltenyi) was added. The cells were then stained with 50 µL of surface staining antibody cocktail for 30 min at RT, incubated with fixation buffer (Fluidigm #201063), and gently vortexed for thorough disruption, followed by incubation for 30 min at RT. After washing twice with Perm Buffer (Fluidigm #201063), the cells were stained with 50 µL of an intracellular staining antibody cocktail and incubated for 30 min at RT. Cells were resuspended in 1.6% PFA at 4°C overnight. After washing with staining buffer (Fluidigm #201063), the cells were resuspended in intercalation solution (Fluidigm #201192A, #201067) for 30 min at RT. The cells were washed with staining buffer (Fluidigm #201068) and resuspended at 0.5 × 10^6^ cells/mL in CAS Buffer (Fluidigm #201241) supplemented with EQ Beads (Fluidigm #201078). Samples were filtered through a 35 µm cell strainer and analyzed using a CyTOF mass cytometer (Fluidigm).

**Table 2:** Antibodies used for Mass Cytometry.

Antigen	Clone	Metal	Manufacturer
CD19	HIB19	142Nd	Fluidigm
CD11b	ICRF44	144Nd	Fluidigm
CD64	10.1	146Nd	Fluidigm
IL-6	MQ213A5	147Sm	Fluidigm
CD14	RM052	148Nd	Fluidigm
CD86	IT2.2	150Nd	Fluidigm
CCR2	K036C2	153Eu	Fluidigm
CD3	UCHT1	154Sm	Fluidigm
CD11c	BU15	159Tb	Fluidigm
CD80	2D10.4	161Dy	Fluidigm
HLA-DR	L243	174Yb	Fluidigm
CCR3	5E8	175Lu	Fluidigm
CD56	NCAM16.2	176Yb	Fluidigm
CD16	3GB	209Bi	Fluidigm
TREM1	TREM-26	172Yb	Fluidigm
CD66	ASL-32	149Sm	Fluidigm
TNFα	Mab11	152Sm	Fluidigm
TLR4	HTA125	158Gd	Fluidigm
CD163	GHI/61	165Ho	Fluidigm
CD32	FUN-2	169Tm	Fluidigm

### Immunohistochemical staining of dermal tissues

Human skin biopsy samples were prepared in 10% buffered formalin, embedded in paraffin, and cut into sections at the Department of Pathology at Kyushu University Hospital. The sections were deparaffinized in xylene, and heat-induced epitope retrieval was conducted using citric acid (pH 6). After blocking with 3% BSA/PBS, the sections were stained with anti-CD14 (rabbit polyclonal, Atlas Antibodies, 1:100) and anti-CEACAM6 (clone 9A6, Abcam, 1:400) primary antibodies. The antibodies were detected with goat anti-mouse IgG (Alexa Fluor 568, Invitrogen, 1:500) and goat anti-rabbit IgG (Alexa Fluor 488, Invitrogen, 1:500) secondary antibodies. Cells were counterstained with DAPI (Dojindo Laboratories, Kumamoto, Japan).

### Isolation and culture of human monocytes

CD14+16- monocytes were negatively isolated using an EasySep Human Monocyte Isolation Kit (STEMCELL Technologies #19359). The cells were cultured in RPMI 1640 medium and stimulated with 10% serum from HCs or patients with SSc in 96-well round-bottom plates at a concentration of 1 × 10^6^ cells/mL for 15 h, and the expression of CEACAM was evaluated using flow cytometry.

### Gene expression microarrays

Total RNA was isolated from the cells using the RNeasy Micro Kit (QIAGEN) according to the manufacturer’s instructions. RNA samples were quantified using an ND-1000 spectrophotometer (NanoDrop Technologies, Wilmington, DE, USA), and the quality was confirmed with a 2200 TapeStation (Agilent Technologies, Santa Clara, CA). The cRNA was amplified, labeled with total RNA using the GeneChip^®^ WT Pico Kit, and hybridized to Thermo Fisher Scientific Clariom™ D Assay, Human, according to the manufacturer’s instructions. All the hybridized microarrays were scanned using an Affymetrix scanner. Relative hybridization intensities and background hybridization values were calculated using the Affymetrix Expression Console™. Raw microarray data were deposited in the Gene Expression Omnibus (GEO) Series GSE207425 database.

### Data analysis and filter criteria

The raw signal intensities of all samples were normalized with the SST-RMA algorithm (gene level) using Affymetrix Expression Console 1.4.1 software. To identify up-or downregulated genes, we calculated Z-scores [Z] and ratios (non-log scaled fold-change) from the normalized signal intensities of each probe for comparison between the control and experimental samples. We then established criteria for regulated genes: (upregulated genes) Z-score ≥ 2.0, ratio ≥ 1.5-fold, (downregulated genes) Z-score ≤ -2.0, and ratio ≤ 0.66.

### ELISA assays

PBMCs were obtained from a patient with SSc. Monocytes were negatively purified using an EasySep Human Monocyte Isolation Kit (STEMCELL Technologies #19359). The CEACAM+ monocyte subset was isolated using CEACAM MicroBeads (Miltenyi). The cells were cultured in RPMI 1640 supplemented with 10% fetal bovine serum (FBS) and 1% penicillin/streptomycin and stimulated with 50 ng/mL LPS in a 96-well round-bottom plate at a concentration of 1 × 10^6^ cells/mL for 6 h. Cytokine levels in the culture medium supernatant were measured using the Human TNF-α Duoset ELISA (R&D Systems) and Human IL-1β ELISA Set II (BD Biosciences) following the manufacturer’s instructions.

### Statistical analysis

Statistical analysis were performed using GraphPad Prism 9. Unpaired t-tests, paired t-tests, Mann–Whitney U tests, or Wilcoxon matched pairs signed rank test were used for comparison of two groups. One-way ANOVA with Tukey’s multiple comparisons were used for comparison of more than two groups. Categorical variables were compared using χ2 or Fisher’s exact test. Non-parametric variable correlation was calculated using Spearman’s rank test. All statistical analysis were performed with two-tailed tests. The data is presented as median ± standard deviation unless otherwise stated. *P* values < 0.05 were considered statistically significant.

## Results

### Increased CEACAM-positive classical monocytes in patients with early SSc

**Figure 1:**
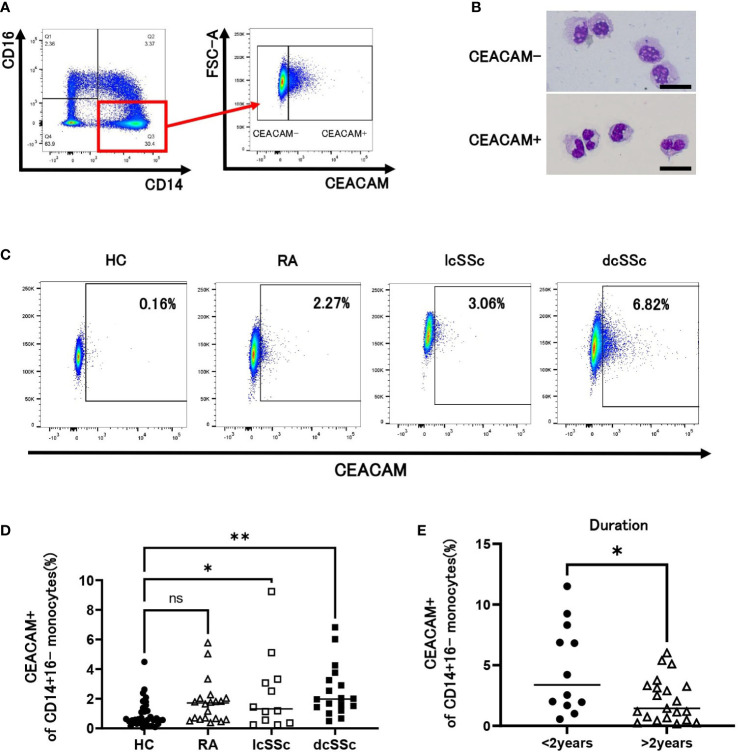
CEACAM expression on CD14+16- classical monocytes. **(A)** Gating strategy for identification of CEACAM+CD14+16- classical monocytes using flow cytometry. **(B)** Giemsa staining for CEACAM-negative (CEACAM-) and CEACAM-positive (CEACAM+) classical monocytes. Sorted CEACAM- and CEACAM+ cells were stained with May-Grünwald Giemsa after cytospin centrifugation. Scale bar represents 20 µm. **(C, D)** CEACAM staining for classical monocytes identified using flow cytometry. Dot plots show CEACAM staining gated on CD14+16- cells from healthy controls (HCs) (n = 34) and patients with rheumatoid arthritis (RA) (n = 20), limited cutaneous systemic sclerosis (lcSSc) (n=12), and diffuse cutaneous systemic sclerosis (dcSSc) (n = 18) analyzed using flow cytometry. Representative data for each group are shown **(C)**. The proportion of CEACAM+ cells in classical monocytes is plotted **(D)**. **(E)** The proportion of CEACAM+CD14+16- monocytes was compared between patients with SSc within 2 years after diagnosis (< 2 years) and those with SSc over 2 years after diagnosis (> 2 years). *P* values were calculated using one-way ANOVA with Tukey’s-multiple comparisons testing between all groups **(D)** and Mann–Whitney U test **(E)**. *P < 0.05, **P < 0.01.

We examined the expression levels of CEACAM on human peripheral classical monocytes (CD14+CD16- cells) by flow cytometry using anti-CEACAM mAb (Clone B1.1) recognizing CEACAM1 (CD66a), CEACAM3 (CD66d), CEACAM5 (CD66e, also known as CEA), CEACAM6 (CD66c). Some classical monocytes expressed CEACAM on their cell surface (**Figure 1A**). Murine CEACAM-positive monocytes have segmented nuclei, such as in neutrophils (21); hence, we performed Giemsa staining for human classical monocytes. As observed in mice, human CEACAM-positive monocytes also presented segmented nuclei (**Figure 1B**). CEACAM-negative conventional monocytes were used as controls (**Figure 1B**). We next investigated the frequency of CEACAM-positive cells in classical monocytes from HCs, patients with SSc and RA using flow cytometry (**Figure 1C**). Patients with lcSSc and dcSSc, but not patients with RA, showed a significantly higher percentage of CEACAM-positive monocytes than HCs (lcSSc 2.4% vs HC 0.92%, *P* < 0.05: dcSSc 2.5% vs HC 0.92%, *P* < 0.01) (**Figure 1D**). Among patients with SSc, those with early SSc within 2 years after diagnosis presented a higher percentage of CEACAM-positive classical monocytes than those over 2 years after diagnosis (3.4% and 1.4% respectively, *P* < 0.05) (**Figure 1E**). In summary, the proportion of CEACAM-positive classical monocytes increased in patients with SSc with short disease duration.

### Increased CEACAM-positive classical monocytes correlated with clinical manifestations in patients with SSc

**Figure 2:**
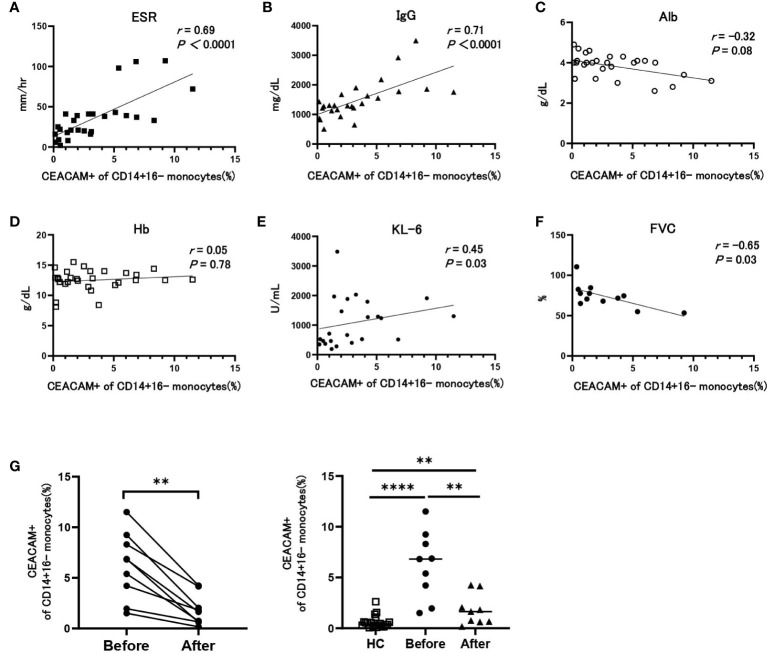
The association between the proportion of CEACAM-positive cells in classical monocytes and clinical parameters in patients with SSc. **(A-F)** Correlation between the proportion of CEACAM+ cells in CD14+16- monocytes and ESR **(A)**, serum IgG **(B)**, serum albumin **(C)**, hemoglobin **(D)**, serum KL-6 **(E)**, and forced vital capacity (FVC) **(F)** in patients with SSc. Correlation coefficients and *P* values were calculated using Spearman’s rank correlation test. **(G)** The proportion of CEACAM+ cells in CD14+16- monocytes before and after immunosuppressive therapies in patients with SSc (n=8) and HCs. *P* values were calculated using a Wilcoxon matched pairs signed rank test. The right figure shows comparisons of the proportion of CEACAM-positive monocytes between HCs and SSc patients before and after therapy with immunosuppressant. *P* values were calculated using a Mann–Whitney U test. **P < 0.01, ****P < 0.0001.

Next, we analyzed the correlation between the proportion of CEACAM+ classical monocytes and clinical parameters of patients with SSc, which were related to disease activity. The percentage of CEACAM-positive monocytes positively correlated with the ESR (r = 0.69) and serum levels of IgG (r = 0.71) (**Figures 2A, B**) and negatively correlated with serum albumin concentration (r = -0.32) (**Figure 2C**); however, it was not associated with hemoglobin (Hb) levels (r = 0.05) (**Figure 2D**). We next examined the relationship of CEACAM-positive monocytes with interstitial lung disease (ILD). The percentage of CEACAM-positive monocytes positively correlated with serum KL-6 concentration, which reflects the disease activity of ILD (r = 0.45) (**Figure 2E**). High levels of serum KL-6 indicate a progressive clinical course of ILD; therefore, we examined the correlation between CEACAM-positive monocytes and forced vital capacity (FVC). The percentage of CEACAM-positive monocytes negatively correlated with FVC (r = -0.67), indicating that patients with SSc with increased CEACAM-positive monocytes presented with progressive pulmonary fibrosis (**Figure 2F**). We next investigated whether the increase in CEACAM-positive monocytes was ameliorated by immunosuppressive therapy. The percentage of CEACAM-positive monocytes significantly reduced after immunosuppressive therapy in all eight patients (**Figure 2G**). In summary, CEACAM-positive monocytes were involved in inflammatory conditions and progressive ILD in SSc and decreased after immunosuppressive therapy in patients with SSc.

### CEACAM6-positive monocytes increased in patients with SSc

**Figure 3:**
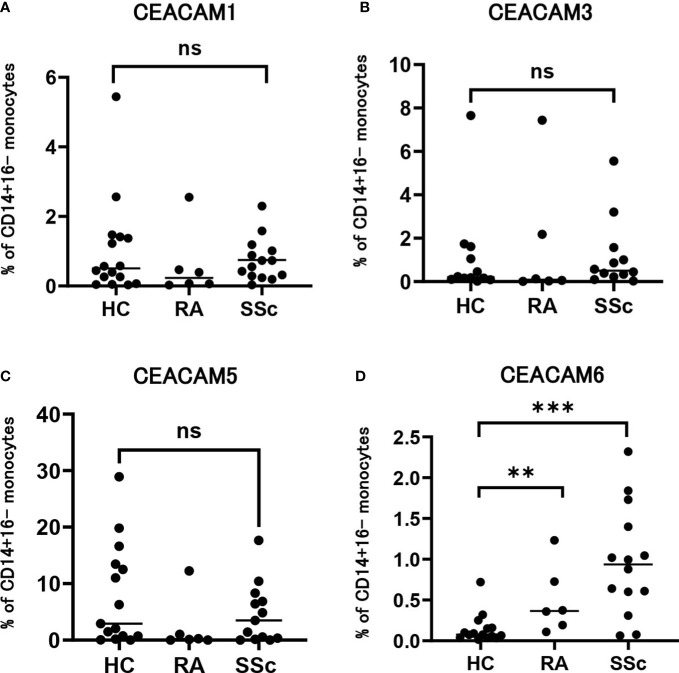
Expression of each subtype of CEACAM on SSc monocytes. **(A-D)** Dot plots show the proportion of positivity of CEACAM1 **(A)**, CEACAM3 **(B)**, CEACAM5 **(C)**, and CEACAM6 **(D)** on CD14+CD16- classical monocytes from HCs and patients with RA and SSc. *P* values were calculated using a Mann–Whitney U test. **P < 0.01, ***P < 0.001. ns, not significant.

CEACAM is encoded by 12 genes in the human genome. CEACAM recognizes bacterial pathogens and CEACAM1, CEACAM3, CEACAM5 and CEACAM6 are pathogen receptors (22). The anti-CEACAM mAb used in this study recognizes these four subtypes of CEACAM. Therefore, we further analyzed the expression levels of each subtype of CEACAM on classical monocytes of patients with SSc and HCs, using subtype-specific mAbs by flow cytometry (**Figures 3A–D**). The proportion of CEACAM1-positive monocytes was lower in patients with RA than HCs; however, the difference was not significant (**Figure 3A**). The proportion of CEACAM3- and CEACAM5- monocytes in patients with SSc were similar to that in HCs (**Figures 3B, C**). The proportion of CEACAM5-positive monocytes decreased in patients with RA compared to that in HCs and patients with SSc. The proportion of CEACAM6-positive monocytes was higher in patients with SSc than that in HCs and patients with RA (**Figure 3D**), but lower than other subtypes. Therefore, the increased rate of CEACAM-positive classical monocytes in patients with SSc was mainly caused by CEACAM6-positive cells.

### SSc serum induced CEACAM6-positive monocytes in HCs

**Figure 4:**
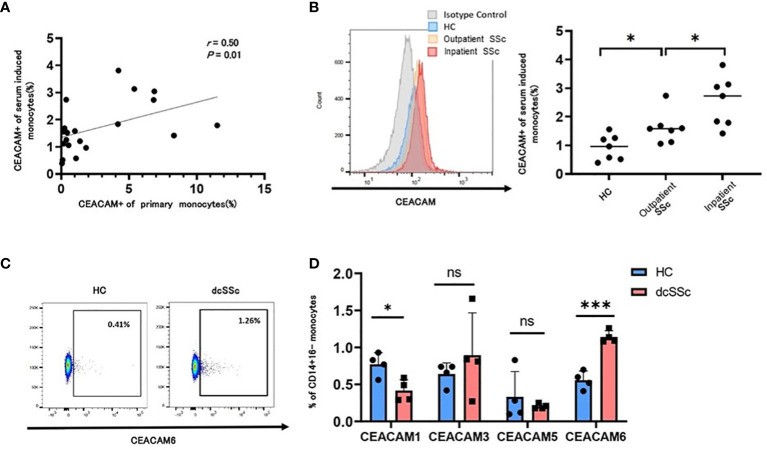
SSc serum induced CEACAM6 expression on classical monocytes. CD14+16- monocytes were isolated from a healthy donor and were treated with serum from HCs (n=7) and patients with systemic sclerosis (n=14) for 15 h. The proportion of CEACAM-positive classical monocytes from patients with SSc who provided serum was also analyzed. **(A)** Dot plots show the correlation between the proportion of CEACAM+ monocytes from a healthy donor treated with SSc serum and that from patients with SSc obtained at the same time as the serum. Statistical analysis was performed using Spearman’s rank correlation test. **(B)** The frequency of serum-induced CEACAM+CD14+16- monocytes was compared among HCs (n=7), inactive SSc (n=7), and active SSc (n=7). Each analysis was performed in duplicates. The left figure shows a histogram of CEACAM. **(C, D)** Data show the frequency of CEACAM1, 3, 5, and 6+ monocytes treated with serum from HCs or patients with dcSSc. Gating of CEACAM6+ monocytes is shown in **(C)** Data are presented as mean ± standard deviation (SD). *P* values were calculated using an unpaired t-test. *P < 0.05, ***P < 0.001.

We next examined whether CEACAM-positive monocytes in patients with SSc were mediated by humoral factors in the serum. Stimulation of monocytes from HCs with SSc serum induced CEACAM-positive monocytes (**Figure 4A**). The proportion of CEACAM-positive monocytes from HCs induced by SSc serum correlated with the proportion of CEACAM-positive classical monocytes in the peripheral blood from each SSc individual (**Figure 4A**). Among those SSc serum, we divided into outpatient and inpatient who required aggressive therapies to observe the relationship between the induction of CEACAM-positive monocytes and the disease severity of SSc. Serum from inpatient induced more CEACAM-positive monocytes than those from outpatient SSc or HCs (**Figure 4B**). Therefore, the effects of SSc serum on the induction of CEACAM expression on monocytes may reflect the disease severity of SSc. Monocytes from dcSSc showed higher percentage of CEACAM-positive cells than those from lcSSc (**Figure 1D**). It was speculated that serum from dcSSc may induce higher percentage of CEACAM-positive monocytes than that from lcSSc, from the result of **Figure 4A**. We then analyzed the expression of each subtype of CEACAM1,3,5, and 6 on monocytes from HCs stimulated with serum from patients with dcSSc or HCs (**Figures 4C, D**). Serum from patients with dcSSc increased the ratio of CEACAM6-positive monocytes and decreased that of CEACAM1-positive monocytes compared to serum from HCs (**Figure 4D**). This tendency was the same as the proportion of each subtype of CEACAM-positive monocytes in the peripheral blood of each SSc individual. These data indicate that humoral factors in the serum of patients with SSc have the potential to induce CEACAM6 expression on classical monocytes.

### CEACAM-positive monocytes present an inflammatory phenotype

**Figure 5:**
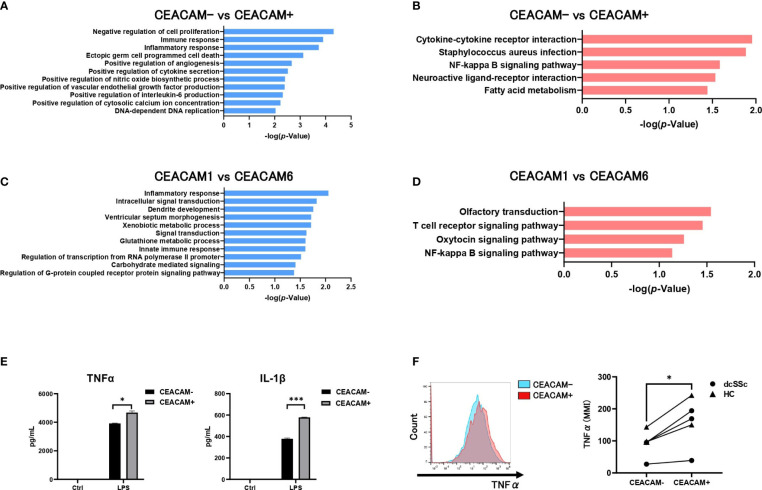
CEACAM-positive monocytes present with an inflammatory phenotype. The GO functional category analysis **(A)** and the KEGG pathway functional classification **(B)** of genes differentially expressed in CEACAM+CD14+16- monocytes and CEACAM-CD14+16- monocytes from a patient with dcSSc. DAVID v6.7 functional annotation bioinformatics microarray analysis software was used to obtain the GO biological process functional category and the KEGG pathway functional classification. Only GO and KEGG pathway terms for categories that showed statistically significant differences are shown (*P*-value ≤ 0.01 in A or *P*-value ≤ 0.05 in B). GO functional category analysis **(C)** and KEGG pathway functional classification **(D)** of genes differentially expressed in CEACAM6+CD14+16- monocytes and CEACAM1+CD14+16- monocytes. Only GO and KEGG pathway terms with *P*-value ≤ 0.05(C) or *P*-value ≤ 0.1(D) are shown. **(E)** ELISA assay for the secretion of TNF-α and IL-1β by CEACAM+CD14+16- and CEACAM-CD14+16- monocytes treated with LPS (50 ng/mL) for 6h. Data are expressed as the mean ± SD, and P values were calculated using an unpaired t-test. **(F)** The intracellular expression of TNF-α was analyzed using CyTOF (n=5). Representative histogram (left) and the Mean Metal Intensity (MMI) (right) are shown. *P*-values were calculated using paired t-tests. *P < 0.05, ***P < 0.001.

To clarify the characteristics of CEACAM-positive monocytes, we performed gene expression microarray analysis of CEACAM-positive and CEACAM-negative classical monocytes from patients with dcSSc. The obtained expression data were analyzed for GO biological process functional categories (**Figure 5A**), and KEGG pathway functional classification (**Figure 5B**). Functional categories or classifications were significantly and differentially expressed between CEACAM-positive and CEACAM-negative monocytes (**Figures 5A, B**). In the GO functional category, immune and inflammatory responses were enhanced in CEACAM-positive monocytes (**Figure 5A**). In the KEGG pathway functional classification, cytokine-cytokine receptor interaction and the NF-κB signaling pathway were enriched in CEACAM-positive monocytes compared to CEACAM-negative monocytes (**Figure 5B**). We further compared CEACAM1-positive monocytes that were decreased in SSc with CEACAM6-positive monocytes that were increased in SSc (**Figures 5C, D**). Gene expression analysis was performed for CEACAM1+CEACAM6- and CEACAM1-CEACAM6+ monocytes. Inflammatory responses and intracellular signal transduction in the GO functional category were more upregulated in the latter than the former (**Figure 5C**). The NF-κB signaling pathways in the KEGG pathway functional classification were enriched in the latter (**Figure 5D**). Therefore, we examined the production of the inflammatory cytokines, TNF-α and IL-1β in CEACAM-positive and CEACAM-negative classical monocytes using ELISA. After LPS stimulation, CEACAM-positive monocytes produced more TNF-α and IL-1β than CEACAM-negative monocytes (**Figure 5E**). In addition, intracellular mass cytometry showed that CEACAM-positive monocytes produced higher levels of TNF-α than CEACAM-negative monocytes in patients with dsSSc and HCs (**Figure 5F**). In addition, cell surface expression of CD86, HLA-DR (M1 marker) and CD163(M2 marker) was analyzed in CEACAM-positive and CEACAM-negative monocytes (**Supplementary Figure 1**). Expression of CD86 was significantly higher on CEACAM-positive monocytes than on CEACAM-negative monocytes. CD163 and HLA-DR tended to be more highly expressed on CEACAM-positive monocytes than on CEACAM-negative monocytes, but without statistical significance. Therefore, CEACAM-positive monocytes were speculated to be more inflammatory than conventional CEACAM-negative classical monocytes.

### CEACAM6-positive monocytes infiltrate the dermis in patients with SSc

**Figure 6:**
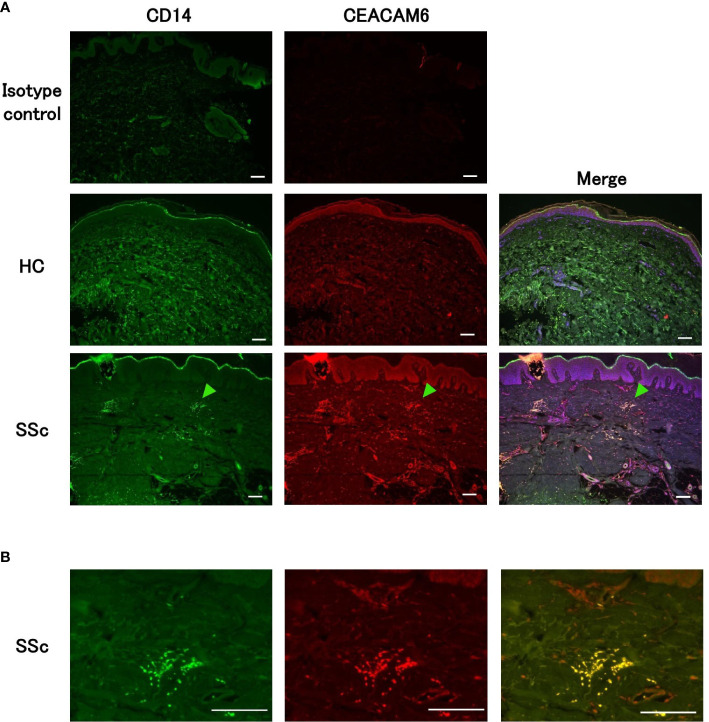
CEACAM6+CD14+ monocytes infiltrate scleroderma skin. **(A)** Representative immunofluorescence of dermal tissues from patients with SSc (n=5) and HCs (n=2). Sections were stained with CD14 (green), CEACAM6 (red), and DAPI (blue). The green arrowhead shows the accumulation of CEACAM6+CD14+ monocytes in the dermis. Sections stained with IgG isotype control for anti-CD14 and anti-CEACAM6 antibodies and detected with corresponding secondary antibodies are also shown as isotype control. **(B)** Magnification images of the arrowhead of part **(A)**. Scale bar represents 100 μm.

Finally, we investigated whether CEACAM6-positive monocytes infiltrated the dermis in SSc. Skin tissues obtained from patients with SSc and controls who were performed random skin biopsy were stained with CD14 and CEACAM6 and analyzed using a fluorescence microscope. CD14-positive monocytes were gathered in the dermis of a patient with SSc but not in that of a control (**Figure 6A**). Some infiltrated CD14-positive monocytes were colocalized with CEACAM6 (**Figures 6A, B**), indicating that CEACAM6-positive monocytes infiltrated the scleroderma dermis.

## Discussion

Here, we show that the proportion of CEACAM-positive classical monocytes in peripheral blood was increased in patients with SSc, especially in those with short disease duration. The frequency of CEACAM-positive monocytes positively correlated with ESR and serum IgG levels. Moreover, patients with SSc with increased CEACAM-positive monocytes showed a high levels of serum KL-6 and decreased %FVC, indicating that CEACAM-positive monocytes are related to the activity and severity of ILD. CEACAM-positive monocytes showed an inflammatory gene expression pattern and produced higher levels of TNF-α and IL-1β than CEACAM-negative monocytes did. Histopathological analysis revealed that CEACAM-positive monocytes infiltrated the dermis in patients with SSc. These data suggest that CEACAM-positive monocytes are involved in the inflammatory conditions in SSc pathogenesis.

In murine bleomycin-induced pulmonary fibrosis, CEACAM1-positive monocytes were involved in lung fibrosis. These cells had segregated nuclei and shared granulocyte characteristics. CEACAM-positive human classical monocytes also showed the bi-lobed segregated nucleus like neutrophils (21). Human monocytes are divided into classical, intermediate, and non-classical monocytes based on cell surface markers CD14 and CD16 (25). CD16 (26) and CEACAM (27) are expressed on neutrophils; thus, neutrophils may be contaminated with CD16-positive intermediate and non-classical monocytes, as examined using flow cytometry and cell sorting. However, classical monocytes are negative for CD16, and most CEACAM-positive classical monocytes presented segregated nuclei; therefore, the CEACAM-positive monocytes analyzed in this study were not contaminated with neutrophils.

Analysis of the subtypes of CEACAM on monocytes revealed that CEACAM6 was predominantly expressed on SSc monocytes, which are not encoded in the rodent genome. The function of CEACAM in human immune cells has mainly been focused on CEACAM1 (28), and that of CEACAM6 is still poorly understood. Mice transgenic for human CEACAM6 do not show perturbation of tissue architecture or tissue homeostasis; hence, CEACAM6 is speculated to become critical during stressful conditions, such as tissue damage and repair (29, 30). Interestingly, CEACAM6 expression is detected in cells of monocytic lineage in human CEACAM6-transgenic mice (29). CEACAM6 contains one IgV-like domain and two IgC2-like domains in its extracellular region. Trans-oligomerization *via* homophilic interactions between the IgV-like domains of CEACAM on neighboring CEACAM-expressing cells supported by IgC2-like domains leads to CEACAM-mediated cell-to-cell adhesion (31). This may contribute to the close contact between CEACAM6-positive monocytes, endothelial cells, and other immune cells that express CEACAM.

CEACAM-positive monocytes increased within 2 years after the diagnosis of SSc and significantly correlated with ESR. Patients with very early SSc treated with corticosteroids showed the improvement in skin fibrosis (32). They are being treated with high-dose methylprednisolone, which potentially prevent early vasculopathy by inhibiting the inflammatory process (33). Corticosteroids relieve the polarization of macrophages towards the classical inflammatory phenotype (34, 35). Patients with SSc are considered to show a more inflammatory phenotype in the early phase than in the late phase (36). Raised ESR in SSc was associated with FVC < 80% predicted and diffusing capacity of the lung (DLCO) < 80%. A significant deterioration in respiratory function tests has been associated with a 2-fold increase in ESR (37). We showed that CEACAM-positive monocytes produced higher levels of TNF-α and IL-1β; therefore, these cells have the potential to cause inflammation. In this study, we could not examine the lung infiltration of CEACAM-positive monocytes because lung samples from patients with SSc-ILD were not available. Therefore, we analyzed scleroderma skin and observed infiltration of CEACAM6-positive monocytes into the dermis. CEACAM-positive monocytes may infiltrate tissues and cause inflammation, resulting in an elevated ESR and ILD. CEACAM is an adhesion molecule that can bind to other CEACAM family members as well as integrin receptors on vascular endothelial cells (30); therefore, CEACAM-positive cells have the potential to migrate out of blood vessels. CEACAM6 is overexpressed in colorectal and gastric cancers and is associated with the adhesion and invasion of tumor cells (38, 39). In murine bleomycin-induced lung fibrosis, CEACAM1-positive monocytes infiltrate the interstitium of the lungs and are involved in lung fibrosis (21). Similarly, patients with SSc with a higher percentage of CEACAM-positive monocytes showed higher levels of serum KL-6 and lower FVC, indicating that patients with increased CEACAM-positive monocytes presented with active and progressive ILD. Although this study could not directly prove that CEACAM-positive monocytes cause lung fibrosis, it is speculated that these cells may have some effects on ILD as shown in mice.

The proportion of CEACAM-positive monocytes also significantly correlated with serum IgG levels. Serum IgG levels with ILD are significantly higher than those without ILD and negatively correlate with %VC and %DLCO in patients with SSc (40). In addition, recent clinical studies have revealed that B cell-targeting therapy using rituximab, an anti-CD20 mAb, is effective for the treatment of progressive fibrosing ILD in SSc (41). The mechanisms by which serum IgG levels reflect pulmonary fibrosis have not yet been clarified; however, auto-antibodies and B cells are involved in the pathophysiology of ILD in SSc. Considering these facts, CEACAM-positive monocytes are speculated to have some direct or indirect effect on IgG-producing cells.

Gene expression microarray analysis showed that CEACAM-positive cells, especially CEACAM6-positive cells, presented with activation of NF-κB pathway. CEACAM6 is a GPI-anchored protein that constitutively localized in lipid rafts (42). Intracellular signaling triggered by epithelial CEACAM leads to activation of PI3-kinase (30, 42). PI3-kinase can activate NF-kB signaling through Akt (43); however, it is difficult to clarify whether CEACAM-positive monocytes in SSc have received intracellular signaling through CEACAM *in vivo*.

SSc serum induced the expression of CEACAM6 on classical monocytes from HCs *in vitro*, indicating that some humoral factors may be involved in the cell surface expression of CEACAM6. Enhanced expression of CEACAM6, which acts as a receptor for adherent-invasive *Escherichia coli*, is also observed at the apical surface of the ileal epithelium in Crohn’s disease (44). The transcriptional regulation of CEACAM6, even in epithelial cells, has not been precisely elucidated. Further studies are required to clarify the mechanisms underlying CEACAM transcription and expression in SSc monocytes.

This study has several limitations. First, the sample size is limited. Larger scale analysis is required for the further investigation of the clinical significance of these cells. Especially, the number of patients analyzed for each subtype of CEACAM was not enough for the evaluation of the correlation between CEACAM6-positive monocytes and clinical parameters of SSc. Second, functions of human CEACAM6-positive monocytes were not investigated in this study because of limited cell numbers in peripheral blood. Lastly, we could not analyze lung tissues to clarify whether CEACAM-positive monocytes infiltrate the site of ILD. It is desirable to analyze lung tissues of SSc-ILD to reveal the mechanisms of CEACAM-positive cells how these cells are involved in the inflammation and fibrosis of lung interstitium.

Collectively, we find that CEACAM-positive cells, especially CEACAM6-positive cells, were increased among classical monocytes in early SSc and were suppressed after immunosuppressive therapy. The proportion of CEACAM-positive monocytes correlated with ESR and progressive ILDs. CEACAM-positive monocytes showed activation of the NFκB signaling pathway, as well as the production of inflammatory cytokines and infiltration into the dermis of SSc. These cells may be useful markers to detect progressive patients requiring therapeutic intervention. In addition, these cells may be one of therapeutic targets in early SSc. Our data provide new insights into the role of CEACAM-positive monocytes in the pathogenesis of SSc.

## Data availability statement

The datasets presented in this study can be found in online repositories. The names of the repository/repositories and accession number(s) can be found below: https://www.ncbi.nlm.nih.gov/geo/query/acc.cgi?acc=GSE207425, GSE207425.

## Ethics statement

The studies involving human participants were reviewed and approved by The Ethics Committee of Kyushu University Hospital. The patients/participants provided their written informed consent to participate in this study.

## Author contributions

KY, HM, and HN participated in study conception and design. KY, SK, YY, and QW participated in acquisition and analysis of data. KY, HM, SK, MA, YK, NO, YA, KA, TH, and HN contributed to the interpretation of results. KY and HM were major contributors in writing the manuscript. All authors contributed to the article and approved the submitted version.

## Funding

This work was supported by JSPS KAKENHI Grant Number JP19164142.

## Acknowledgments

We would like to thank all SSc patients and HC who participated in this study. We also thank Dr. Kaori Yasuda (Cell Innovator, Inc.) for technical support and fruitful discussion.

## Conflict of interest

The authors declare that the research was conducted in the absence of any commercial or financial relationships that could be construed as a potential conflict of interest.

## Publisher’s note

All claims expressed in this article are solely those of the authors and do not necessarily represent those of their affiliated organizations, or those of the publisher, the editors and the reviewers. Any product that may be evaluated in this article, or claim that may be made by its manufacturer, is not guaranteed or endorsed by the publisher.

## Supplementary material

Supplementary Figure 1 Expression of M1 and M2 marker on CEACAM-positive monocytes. The expression of CD86, HLA-DR, and CD163 on CEACAM-positive and CEACAM-negative monocytes was analyzed using CyTOF (n=5). The Mean Metal Intensity (MMI) of each molecule in CEACAM-positive and CEACAM-negative monocytes is indicated. *P* values were calculated using paired t-tests. ***P* < 0.01.
