# Supplementary figures and images for: CEACAM 1, 3, 5 and 6 -positive classical monocytes correlate with interstitial lung disease in early systemic sclerosis
Source: Front Immunol. 2022 Oct 20;13:1016914. doi: 10.3389/fimmu.2022.1016914 (PMC9632165; doi:10.3389/fimmu.2022.1016914)

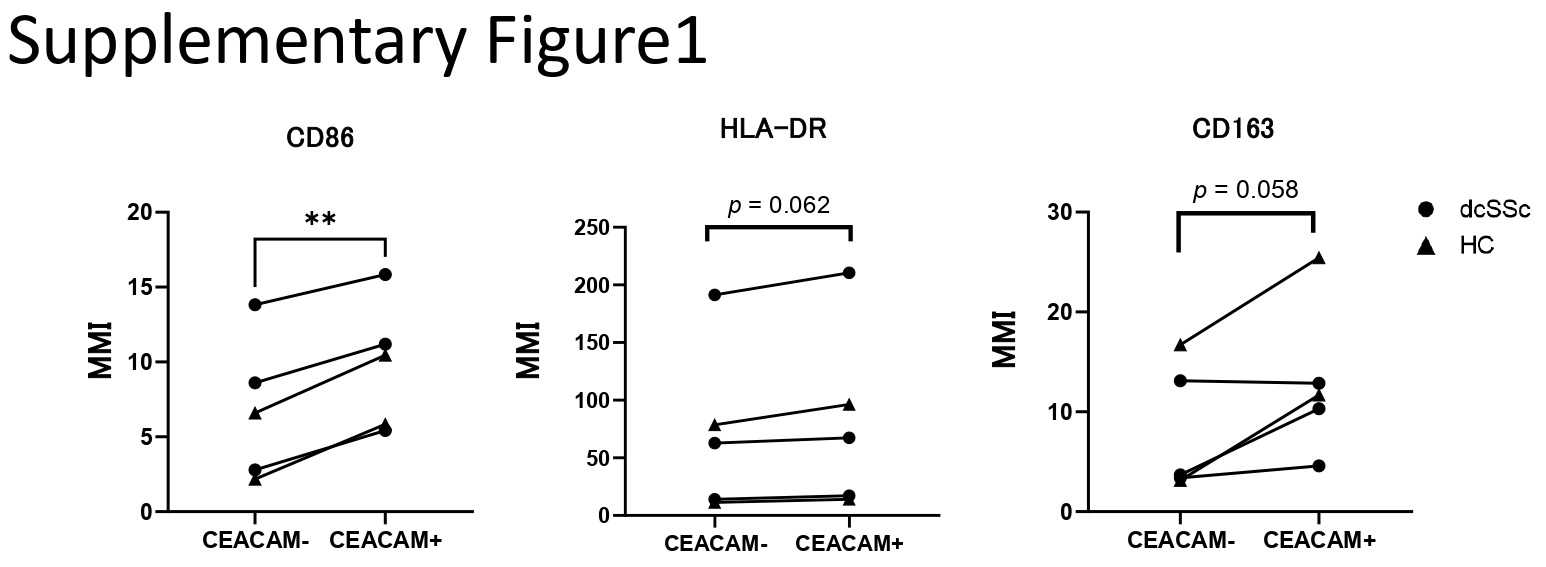

Supplement: Supplementary Figure 1 — Expression of M1 and M2 marker on CEACAM-positive monocytes. The expression of CD86, HLA-DR, and CD163 on CEACAM-positive and CEACAM-negative monocytes was analyzed using CyTOF (n=5). The Mean Metal Intensity (MMI) of each molecule in CEACAM-positive and CEACAM-negative monocytes is indicated. P values were calculated using paired t-tests. **P < 0.01. [file Image_1.jpeg]
